# A transgenic ginseng vaccine for bovine viral diarrhea

**DOI:** 10.1186/s12985-015-0301-9

**Published:** 2015-05-07

**Authors:** Yugang Gao, Xueliang Zhao, Chao Sun, Pu Zang, He Yang, Ran Li, Lianxue Zhang

**Affiliations:** College of Traditional Chinese Medicine, Jilin Agricultural University, ChangChun, 130118 China; College of AnimCal Science and Technology, Northwest A & F University, Yang Ling, 712100 China

**Keywords:** Bovine viral diarrhea virus, Transgenic ginseng vaccine, E^rns^

## Abstract

**Background:**

Bovine viral diarrhea virus (BVDV) infections are endemic in cattle populations worldwide and cause major economic losses. Thus, an effective vaccine is needed against the transmission of BVDV. The glycoprotein E^rns^ is one of the envelope proteins of this virus and shows BVDV-related immunogenicity. Here, we report the use of *Panax ginseng* as an alternative production platform for the expression of glycoprotein E^rns^ via *Agrobacterium*-mediated transformation.

**Result:**

Polymerase chain reaction (PCR) and reverse transcription (RT)-PCR analyses showed that pBI121-E^rns^ was stably integrated into the chromosome of transformants. ELISA assay and Western blot analysis confirmed the antigenicity of plant-derived E^rns^ glycoprotein. Immunogenicity was evaluated subcutaneously in deer using a soluble protein extract of dried transgenic ginseng hairy roots. Specific humoral and cell-mediated immune responses against BVDV were detected following immunization.

**Conclusion:**

These results demonstrated that the E^rns^ glycoprotein could be expressed in ginseng hairy roots and that plant-derived glycoprotein E^rns^ retained its antigenicity and immunogenicity.

## Introduction

Bovine viral diarrhea virus (BVDV) is a positive-stranded RNA virus of the Flaviviridae family [1,2] and an important cause of economic loss in herds worldwide. BVDV infections can have several consequences such as acute infection, diarrhea, fertility problems, and fatal mucosal diseases, the incidences of which have been rising in recent years [3,4]. Some reports have demonstrated that BVDV infection could damage the immune system of the infected animals and make them more susceptible to other diseases. In addition, BVDV can be transmitted between a variety of animals such as cattle, sheep, and whitetail deer [5-8] and the presence of BVDV in other domestic species might be relevant to its epidemiology [9]. It has been reported that BVDV infection rates in young deer reached 60% ~ 86.7% in some areas of China [10], which caused economic losses in the sika deer industry due to the high mortality and fetal infections associated with the disease. In our previous study, a new single strain of BVDV, named CCSYD, was isolated and verified from sika deer [11]. The BVDV infection in sika deer is a serious concern and an effective strategy is needed to fight against BVDV transmission.

Vaccines have been shown to be effective tools for use in the eradication of economically important animal pathogens. There are several available commercial vaccines against bovine viral diarrhea (BVD) but these show irregular performances. The low immunogenicity of inactivated BVDV vaccines often leads to immune failure. Although modified live vaccines provide certain protection against homologous strains, the intrinsic risk of virulence reversion remains a concern [12,13]. Thus, the use of recombinant subunit vaccines has been proposed as an alternative to overcome these difficulties [14].

BVDV genomes are translated and processed into eleven mature proteins which include a capsid protein (C), one N-terminal protease (Npro), three envelope glycoproteins (E1, E2, and E^rns^), a protein of 7 kDa (p7), and five non-structural proteins (NS2-3, NS4A, NS4B, NS5A, and NS5B). After infection or vaccination, cattle produce antibodies against the three envelope proteins (E1, E2, and E^rns^) and against a non-structural protein (NS2-3) [15]. Glycoprotein E2 is the major target of the neutralizing antibodies in BVDV-infected hosts [16,17]. However, the highly variable sequence of the E2 protein often leads to immunization failure [18,19].

The E^rns^ glycoprotein is one of the virus structural proteins. Several studies have indicated that some of the immunologically relevant E^rns^ epitopes are conserved among different BVDV isolates, and few differences are found in E^rns^ amino acid composition among different pestiviruses [20,21]. As a conserved protein, BVDV E^rns^ has been used as an antigen for the serological detection of BVDV [22]. BVDV E^rns^ expressed in prokaryotic systems also produces neutralizing antibodies; however, they are produced at low titers that are unable to efficiently neutralize the virus. This was attributed to the misfolding of E^rns^ when expressed in prokaryotes [23].

Because eukaryotic expression can maintain correct folding and glycosylation of proteins, eukaryotic expression has become a research focus in the study of subunit vaccines. In our previous study, Gao et al. [24] successfully constructed a prokaryotic expression vector PVAX1-E0 and confirmed that the recombined PVAX1-E0 could produce specific humoral and cellular immune responses in rabbits. However, the subunit vaccines only offered short-term immunity. Transgenic plants are new eukaryotic expression-delivery systems that have become attractive bioreactors in the production of high-value medical peptides and proteins [25]. Plant-based vaccines offer several advantages over traditional vaccines such as ease of delivery, mucosal efficacy, safety, rapid scalability, and low cost. To date, several plant species have been used as antigen-delivery systems for subunit vaccines [26,27]. For example, truncated glycoprotein BVDV E2 has been expressed in *Nicotiana tabacum* leaves and subsequently showed high reactivity in virus neutralization tests [28].

Another way to improve the immune activity of vaccines is the use of an adjuvant. Vaccine adjuvants can stimulate the immune system to increase the specific antibody response. *Panax ginseng* C.A. Meyer, commonly known as ginseng, has been used as a medicinal plant in East Asia for over 2000 years [29,30]. The major function of ginseng is the stimulation of natural resistance against infections [31]. Recently, research has shown that extracts of ginseng can exert a number of effects on the immune system such as improvement of the phagocytic activity of macrophages, lymphocyte proliferation enhancement, cytokine production stimulation, and increased activity of neutrophils, CD4^+^ T cells, and natural killer cells [32-34].

## Results

### Genetic analysis of transformed plants

**Figure 1:**
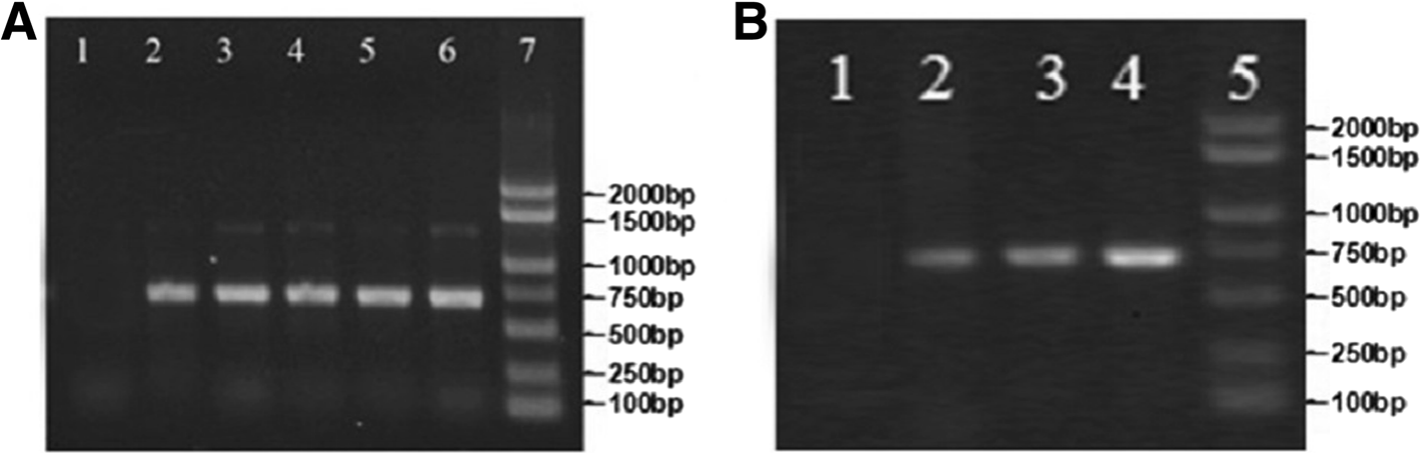
Genetic identification of transgenic ginseng hairy roots. Genomic DNA and total RNA were extracted from regenerated seedling for PCR **(A)** and RT-PCR **(B)** identification. DNA gel blot analysis was used to detect the pBI121-E^rns^ transgene in transgenic ginseng hairy roots. **(A)** Lanes 3, 4, 5, and 6 show the E^rns^ gene fragments (total genomic DNAs extracted from the leaves of different transgenic ginseng plants). Lane 1 is a negative control (total genomic DNAs extracted from the leaves of different untransformed wild-type ginseng plants). Lane 2 shows the positive control by using pBI121-E^rns^ plasmid DNA as PCR template and lane 7 shows the molecular mass markers. **(B)** RT-PCR analysis. Lane 1: RT-PCR product from untransformed ginseng. Lane 2: PCR product from pBI121-E^rns^ plasmid. Lanes 3–4: RT-PCR product from different transgenic ginseng hairy roots. Lane 5: DNA molecular mass marker.

Transgenic ginseng hairy roots were successfully obtained. After the isolation of genomic DNA and total RNA from transgenic hairy roots, 706 bp long bands were detected using polymerase chain reaction (PCR) and reverse transcription (RT)-PCR in all ginseng hairy roots except in the negative control groups, which confirmed the stable integration of the expression vector pBI121-E^rns^ into the chromosome of the transformants (Figure 1A and B).

### E^rns^ protein expression in transgenic ginseng hairy roots

**Figure 2:**
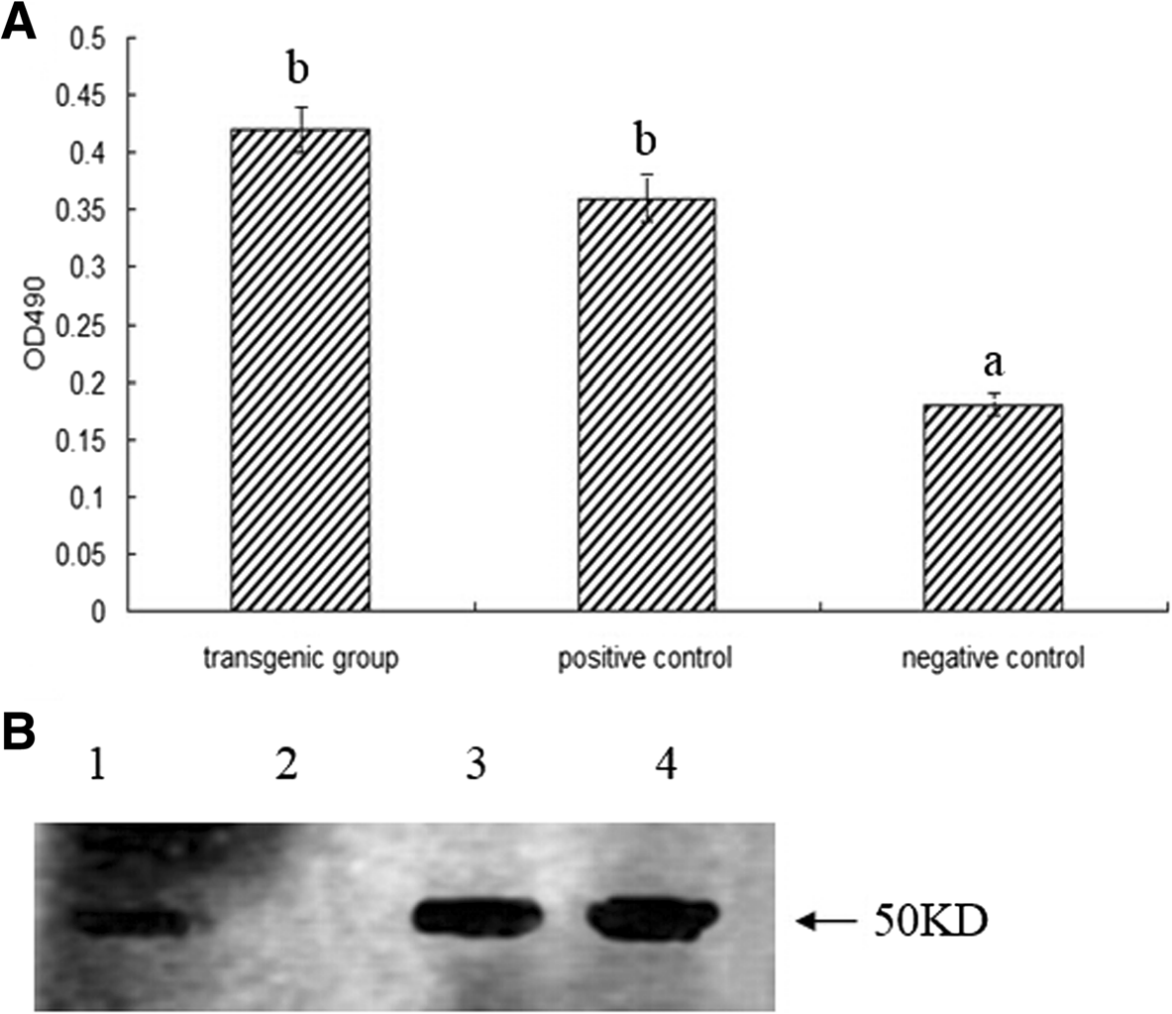
Protein expression analysis in transgenic *Panax ginseng* extract. **(A)** ELISA was used to determine the presence of antigen E^rns^ in extracts from transgenic ginseng hairy roots. The ELISA protocol is described in the Materials and methods section. Lane 1: recombinant proteins extracted from transgenic ginseng hairy roots. Lane 2: positive control (E^rns^ protein expressed in baby hamster kidney (BHK)-21 cells. Lane 3: negative control (proteins extracted from untransformed ginseng hairy roots). ^a–b^ The difference between the negative control (proteins extracted from untransformed ginseng hairy roots) and other groups is significant (*P* < 0.05). The result showed that the soluble proteins from the transgenic group had immune reactivity against anti-BVDV antiserum and the OD_490_ values of the transgenic groups were significantly higher than those of the negative controls. **(B)** Western blot analysis showing the immune activity of E^rns^ protein expressed in transgenic ginseng hairy roots*.* In the transgenic ginseng hairy roots group (Lane 1) and the positive control [Lane 3, E^rns^ protein expressed in baby hamster kidney (BHK)-21 cells] a specific band of 50 kDa was detected. Lane 2, negative control (proteins extracted from untransformed ginseng hairy roots).

To determine whether E^rns^ protein was expressed in transgenic ginseng hairy roots, first, enzyme-linked immunosorbent assay (ELISA) was carried out to detect the antigen presence in the total soluble proteins from transgenic ginseng hairy roots. The result showed that the soluble proteins from the transgenic group had immune reactivity against anti-BVDV antiserum and the OD_490_ values of the transgenic groups were significantly higher than those of the negative controls (Figure 2A), which implied that E^rns^ protein was expressed and accumulated in transgenic *Panax ginseng.*

To further confirm the immunogenicity of the soluble proteins from the transgenic group against anti-BVDV antiserum, Western blot analysis was carried out. The result showed that a specific signal was detected in the total soluble proteins from the selected transgenic plants after immunoblotting with anti-BVDV antiserum (Figure 2B), while no signal was observed in the untransformed groups. The result further confirmed that the E^rns^ proteins expressed in transgenic ginseng hairy roots were immunoreactive to anti-BVDV antiserum.

### Detection of deer serum antibody and cellular immune level

**Figure 3:**
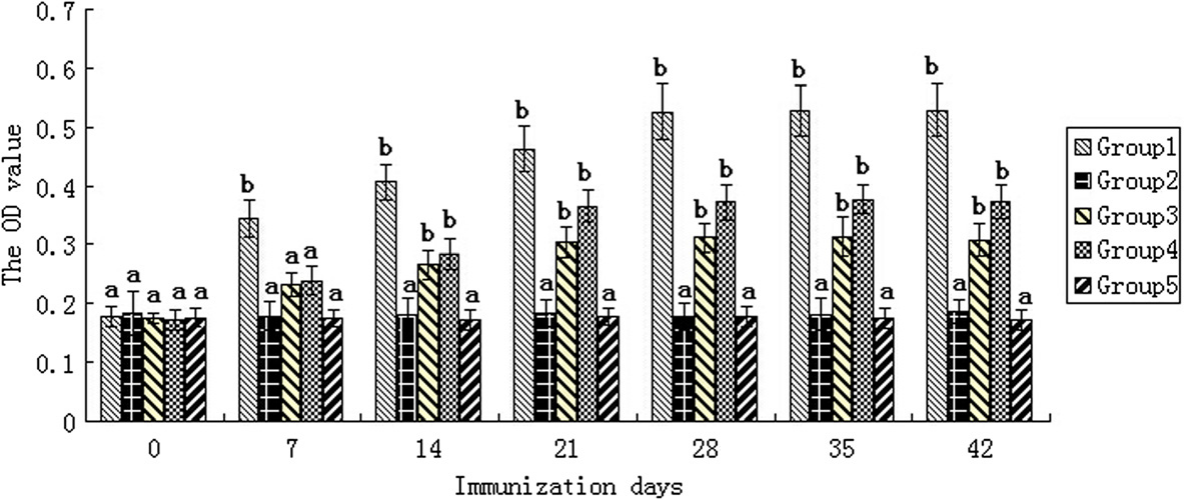
Specific humoral response in deer following vaccination. Specific humoral response in deer following vaccination. The deers were immunized subcutaneously with an aqueous extract of transgenic ginseng hairy roots (group 1), an aqueous extract of untransformed ginseng hairy roots (group 2), inactivated BVDV vaccine (group 3), inactivated BVDV vaccine and untransformed ginseng hairy roots extract (group 4), and saline (group 5). Serum samples used to assess the presence of BVDV specific antibody were collected on days 0, 7, 14, 21, 28, 35, and 42 after primary immunization and detected at 490 nm. The OD values increased with time after immunization in all vaccinated groups, except in the control groups (groups 2 and 5). The result reveals that the transgenic plant vaccine (group 1) could produce a specific humoral immune response in deer. ^a–b^ The difference between the negative control (group 5) and other groups is significant (*P* < 0.05).

Serum samples were used to evaluate antibody levels of immunized deer. As shown in Figure 3, the OD values increased with time after immunization in all vaccinated groups, except in the control groups (groups 2 and 5). Antibody level from groups 3 and 4 increased continuously, rising to an apex 11 days after the second inoculation. Vaccinated animals from group 1 showed high antibody level which reached a peak 18 days after the second inoculation. No significant increases in antibody level were detected in the negative control groups (*P* > 0.05). The result revealed that the transgenic plant vaccine (group 1) could produce a specific humoral immune response in deer.

**Figure 4:**
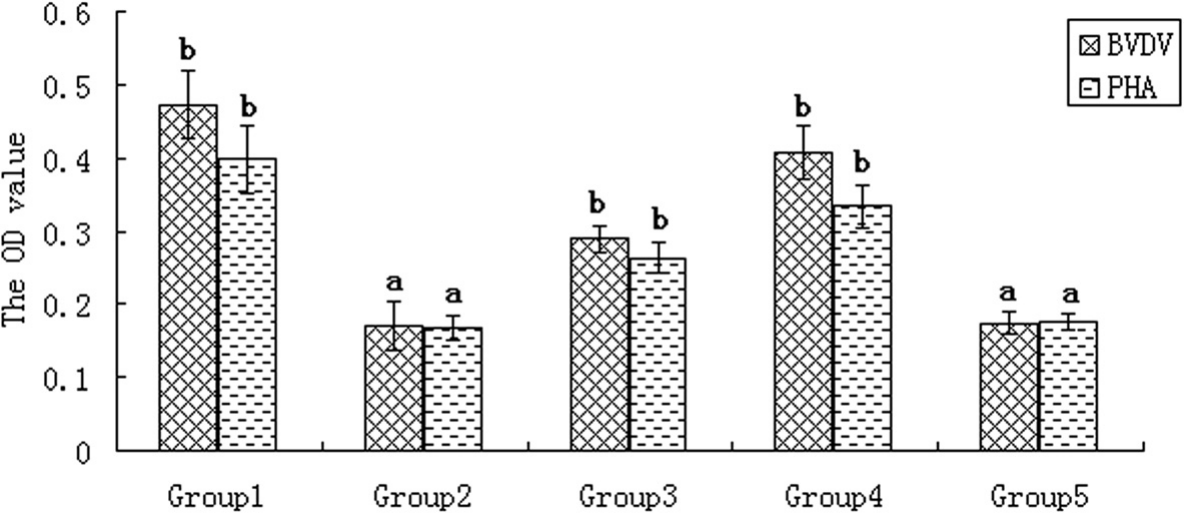
Lymphocyte blastogenesis assay. Blood was collected from deer on the 42nd day after immunization. Group 1: transgenic ginseng vaccine. Group 2: untransformed ginseng vaccine. Group 3: inactivated BVDV vaccine. Group 4: inactivated BVDV + untransformed ginseng vaccine. Group 5: negative control. The result showed that there was a significant improvement in lymphocyte proliferation in the group treated with the transgenic hairy roots vaccine and suggested that E^rns^ protein expressed in transgenic ginseng hairy roots could prolong cell-mediated immune responses against BVDV antigens. ^a–b^ The difference between the negative control (group 5) and other groups is significant (*P* < 0.05).

The sera collected at the time of immunization and on 42 days post-immunization were used to detect cell-mediated immune responses. The results showed that there was a significant improvement in lymphocyte proliferation in the group treated with the transgenic hairy roots vaccine, inactivated BVDV vaccine, and inactivated BVDV vaccine group + untransformed ginseng hairy roots extract upon restimulation with BVDV antigens (Figure 4). No specific proliferation was detected in the control groups (groups 2 and 5) (Figure 4). These results refer to phytohemagglutinin (PHA)-induced lymphocyte proliferation and suggested that E^rns^ protein expressed in transgenic ginseng hairy roots could prolong cell-mediated immune responses against BVDV antigens.

## Discussion

Developing effective and inexpensive vaccines is vital for the protection of animals against various infectious diseases, particularly those that are highly transmissible. Owing to the development of genetic molecular biology and plant biotechnology, plants have emerged as a new platform for the production and delivery of antigen proteins in the form of plant-based vaccines. Using plant expression systems to produce vaccines offers several advantages over mammalian cells and yeast systems such as easy storage and transportation, low cost, and enhanced safety because the risks associated with human pathogen contamination and needle-based delivery are avoided. Vaccines produced in crop plants, when administered orally, can elicit both systemic and mucosal protective immunities.

*Panax ginseng* C.A. Meyer, known for a thousand years, has long been used as a valuable traditional Chinese medicine. It has been reported that ginseng contains many biologically active components such as ginsenosides, polyacetylenes, acidic polysaccharides, ginseng proteins, and phenolic compounds [35]. Ginsenosides and polyacetylenes are the primary components of Asian ginseng. Some reports have indicated that ginsenosides and polyacetylenes show high immunoregulatory activity [36] while other investigations have also demonstrated that using ginseng as a vaccine adjuvant can stimulate the immune system to exert an increased specific antibody response [37].

However, there are still no reports regarding the use of ginseng as a platform for antigen expression. As a perennial herb, *Panax ginseng* is difficult to regenerate quickly and genetically transform. In this study, we have successfully induced the production of transgenic ginseng hairy roots with *Agrobacterium rhizogenes* containing the E^rns^ gene. The glycoprotein E^rns^ was expressed and accumulated in transgenic ginseng hairy roots. Ginseng extracts used as vaccine adjuvants significantly improved the immune activity of the E^rns^ subunit vaccine. Furthermore, plant-derived glycoprotein E^rns^ has the ability to generate an immune response in sika deer. This study provides a new method by which a protein with weak immunogenicity can be used as a transgenic plant vaccine.

## Materials and methods

### Equipment and materials

The plasmid pBI121-E^rns^ and *Agrobacterium rhizogenes* A4 strains were provided by our laboratory. Restriction enzymes, Taq DNA polymerase, TriPure Kit, and T4 ligase were purchased from TaKaRa Biotechnology Co. (Dalian, China) and used to construct the recombinant plasmid pBI121-E^rns^. Ginseng (*Panax ginseng* C.A. Meyer) was cultured in the Jilin Agricultural University (China).

### Ethics statement

All deer were obtained from the DongDa Deer Industry Co., Ltd. Deer care and maintenance was carried out at the sika deer farm of the Jilin Agricultural University and permission to undertake this work was granted by the Management Bureau of Animal Husbandry in Jilin Province (Shaoxian Liu, Director). All deer were maintained as per the directive rules of raising sika deer (2012) and were approved by DongDa Deer Industry Co., Ltd. Animal welfare and experimental procedures were carried out in accordance with the Protocol Guide GB/T 14926.50-2001, which was approved by the Standardization Administration of the People’s Republic of China. The research was also approved by the animal ethics committees of the Jilin Agriculture University and the National Deer Industry Association of China Animal Agriculture Association (CAAA). All deer were anesthetized prior to sampling. The blood samplings were performed by veterinarians, biologists, or technicians with previous training and experience in these procedures. We collected up to 2 mL of blood via the jugular vein. All surgery was performed under sodium pentobarbital sedation and all efforts were made to minimize suffering. Finally, none of the deer were sacrificed.

### Plasmid construction

Total RNA from MDBK cells infected with the CCSYD BVDV strain (belonging to BVDV 1b) was isolated and reverse transcription was performed using hexa-random primers (Biodynamics SRL, Argentina). The E^rns^ open reading frame was amplified using the forward primer (5’-CCG GAT CCA TGG AAA ACA TAA CAC AGT GG-3’, *BamH*I site underlined) and the reverse primer (5’-GCG AGC TCT TAA GCG TAT GCT CCA AAC CAC GT-3’, *Sac*І site underlined). The PCR product was digested with *BamH*I and *Sac*І and inserted into the plant expression vector pBI121, which was digested with the same enzymes to finally create the recombinant plasmid pBI121-E^rns^. The obtained recombinant vector was introduced into *Agrobacterium rhizogenes* A_4_ strain by electroporation using the procedure previously described [38].

### Cultivation of Agrobacterium rhizogenes A_4_ strain

*Agrobacterium rhizogenes* A_4_ strain cells containing pBI121-E^rns^ were added to YEB solid culture medium and activated three times at 27°C. A single colony isolated from the Petri dish was then inoculated into 25 mL YEB liquid culture medium and grown overnight at 27°C shaking at 110 r min^−1^.

### Preparation of explants

The rhizomes of two-year old ginsengs were thoroughly cleaned with running tap water for 1–2 h, surface sterilized, and then disinfected by a brief rinse (30 s) in 75% ethanol (*v*/*v*). The cleaned explants were finally treated with HgCl_2_ (0.1%) for 6–8 min under aseptic conditions and washed 5 times with sterile distilled water to remove traces of HgCl_2_. Then, the sterile rhizomes were cut into 2–3 mm slices and cultured on Murashige and Skoog (MS) medium for 2 days before the transformation experiments.

### Transformation procedure

*Agrobacterium rhizogenes*-mediated transformation was carried out as described in our previous study [39]. After cultivation for one or two months, roots started to appear at the infection sites and single roots were subsequently picked out and placed on fresh MS media with kanamycin (500 mg L^−1^) to eliminate any bacteria. Hairy roots with no bacterial contamination were cultured on hormone-free half-strength MS solid medium in the dark at 26°C. Hairy root lines were sub-cultured at 4 week intervals. Transgenic hairy roots of *Panax ginseng* were verified by PCR and RT-PCR using the forward primer 5’-CCG GAT CCA TGG AAA ACA TAA CAC AGT GG-3’ and the reverse primer 5’-GCG AGC TCT TAA GCG TAT GCT CCA AAC CAC GT-3’. Ginseng explants were also infiltrated with a wild-type *Agrobacterium rhizogenes* A4 strain to obtain negative controls.

### Protein extraction

Protein isolation was conducted using 0.5 g transgenic hairy roots that were quickly ground in liquid nitrogen. The resulting powder was resuspended in 1 mL extraction buffer (125 mM Tris-Cl pH 6.8, 4 mol L^−1^ urea, 4% SDS, and 5% β-mercaptoethanol) and the resulting mixture centrifuged at 12,000 × *g* for 20 min at 4°C. The protein concentration in 1 mL of the supernatant was detected by the Bradford protein assay [40] using bovine serum albumin (BSA) as a standard.

### Enzyme-linked immunosorbent assay (ELISA)

ELISA was employed to detect the expression of glycoprotein E^rns^ in transgenic hairy roots [41]. Briefly, the extracts were incubated in the ELISA plate at 4°C overnight, followed by 3 washes with PBST, and blocking with 10% horse serum for 1 h at 37°C. After another 3 washes with PBST, anti-BVDV antiserum (1:20 dilution) was used as primary antibody. Then, the plates were incubated for 1 h at 37°C with horseradish peroxidase (HRP) conjugated rabbit anti-bovine IgG (1:5,000 dilution) as secondary antibody. The reaction was stopped after 5 min with 2 M H_2_SO_4_ (50 μL well^−1^) and the absorbance was examined using an ELISA reader at 490 nm.

### Western blot analysis

The soluble proteins prepared from control and transgenic plants were analyzed by Western blot as previously described [41]. The extracted total soluble proteins from fresh transgenic hairy roots were subjected to electrophoresis on 12% sodium dodecyl sulfate polyacrylamide gel (SDS-PAGE) and transferred to a nitrocellulose membrane (GE Healthcare, USA). Western blotting was carried out using anti-BVDV antiserum (1:100 dilution) and HRP-conjugated rabbit anti-bovine IgG (1:5,000 dilution, Southern Biotechnology, USA) as primary and secondary antibodies, respectively. The signals were developed using a SuperSignal West Pio Luminol kit (Pierce, Rockford, USA).

### Immunization schedule

Forty healthy 1-month-old male sika deers were randomly divided into 5 groups (8 deer per group). The air-dried transgenic and untransformed ginseng hairy roots were soaked in physiological saline solution for 24 h. Then, the aqueous extracts were filtered through 0.22 μm microporous membranes for sterilization. Group 1 was immunized subcutaneously (s.c.) with 1 mL aqueous extract from transgenic ginseng hairy roots (equivalent to 0.1 g transgenic ginseng hairy roots). Group 2 was immunized s.c. with 1 mL aqueous extract from untransfromed ginseng hairy roots with the same dose as group 1. Group 3 was immunized s.c. with 100 TCID_50_ (median tissue culture infective dose) of inactivated BVDV vaccine (purchased from Chinese Veterinary Drug Control Room), while group 4 was immunized s.c. with 100 TCID_50_ of inactivated BVDV vaccine and 1 mL aqueous extract from untransformed ginseng hairy roots (equivalent to 0.1 g transgenic ginseng hairy roots). Group 5 was injected s.c. with 1 mL saline as a negative control. The second immunization was carried out in all groups on the 14th day after the initial immunization. Blood samples were collected at the time of immunization (day 0) and on the 7th, 14th, 21st, 28th, 35th, and 42nd day after the first immunization.

### Determination of specific antibodies in immunized deer

Indirect ELISA was carried out to test the presence of the specific antibody in the sera from immunized deer. The blood taken from the sera from deer was diluted (1:40) in coating buffer (pH 8.0) and added to microtiter plates (100 μL well^−1^). The microtiter plates were incubated for 2 h at 37°C. After removing the liquid, the plates were washed 3 times with PBST, and blocked with 10% horse serum for 2 h at 37°C. Then, 100 μL of soluble whole virus antigen (containing 100 μg viral proteins) extracted from the C_24_V BVDV (purchased from the China Institute of Veterinary Drug Control) was added to the plates and incubated for 2 h at 37°C. After this, plates were washed and blocked as mentioned above. The absorbance was examined using an ELISA reader at 490 nm.

#### Lymphocyte blastogenesis assay

Peripheral blood mononuclear cells were isolated from anti-coagulated jugular blood and the cell viability was assessed with a trypan blue dye exclusion assay. A survival rate >95% was considered for quantification. Cell suspensions were placed into 96-well round-bottom plates at a concentration of 5 × 10^6^ cells well^−1^ (100 μL). Then, the plates were incubated with or without BVDV (10^4^ TCID50 well^−1^). The MTT method was carried out to detect cell viability [41]. The absorbance was examined at 570 nm.

#### Statistical analysis

Multiple group comparisons were performed using one way analysis of variance (ANOVA) followed by Tukey’s test in order to detect intergroup differences. GraphPad Prism software (Version 5.0; GraphPad Software, Inc., San Diego, CA) was used to perform the statistical analysis. A *P*-value of <0.05 was considered statistically significant.

## Conclusions

In summary, glycoprotein E^rns^ of BVDV can be effectively expressed in transgenic ginseng hairy roots and used as an antigen source for a possible vaccine against BVDV infection. Whether viral envelope proteins can improve the biosynthesis and accumulation of other kinds of secondary metabolites would be the subject of future research, and studies to investigate these and other aspects are currently underway in our laboratory.
